# Trisomy 5p with bilateral congenital diaphragmatic hernia: a case report

**DOI:** 10.1186/s13256-021-02710-y

**Published:** 2021-03-10

**Authors:** Noriyuki Nakamura, Takafumi Ushida, Yoshinori Moriyama, Kenji Imai, Tomoko Nakano-Kobayashi, Satoko Osuka, Maki Goto, Hiroaki Kajiyama, Hideyuki Asada, Masahiro Hayakawa, Tomomi Kotani

**Affiliations:** 1 Department of Obstetrics and Gynecology, Nagoya University Graduate School of Medicine, 65 Tsurumai-cho, Showa-ku, Nagoya, 466-8550 Japan; 2 Department of Obstetrics and Gynecology, Fujita Health University School of Medicine, 1-98 Dengakugakubo, Kutsukake-cho, Toyoake, Aichi 470-1192 Japan; 3 Division of Perinatology, Center for Maternal-Neonatal Care, Nagoya University Hospital, 65 Tsurumai-cho, Showa-ku, Nagoya, 466-8560 Japan; 4 Department of Neonatology, 3-35 Michishita-cho, Nakamura-ku, Japanese Red Cross Nagoya Daiichi Hospital, Nagoya, 453-8511 Japan; 5 Division of Neonatology, Center for Maternal-Neonatal Care, Nagoya University Hospital, 65 Tsurumai-cho, Showa-ku, Nagoya, 466-8560 Japan

**Keywords:** Diaphragmatic hernia, Macroglossia, Polyhydramnios, Talipes equinovarus

## Abstract

**Background:**

Bilateral congenital diaphragmatic hernia (CDH) is very rare. A few studies have reported the pathogenic role of 5p in CDH.

**Case presentation:**

A 23-year-old primigravida Japanese woman was referred for the following abnormal findings at 33 weeks of gestation: polyhydramnios, macroglossia, talipes equinovarus, and levocardia. A marker chromosome was detected by amniocentesis. Fluorescence in situ hybridization with whole chromosome paint 5 and nucleolus organizer region probes confirmed its origin from chromosome 5 and an acrocentric chromosome. The karyotype of the fetus was diagnosed as 47, XY, +mar. ish +mar(WCP5+). At 39 + 5 weeks, a 2462 g male infant was delivered, with a specific facial configuration. Bilateral CDH, hypoplasia of the corpus callosum, atrial septal defect, and hypothyroidism were also detected in the baby. The karyotype of the peripheral blood was consistent with that of the amniocentesis.

**Conclusion:**

Genes coded on 5p might be associated with the pathogenesis of CDH; however, further investigation is required.

## Background

Trisomy 5p is a rare chromosomal abnormality with the following clinical features: facial dysmorphism, brain abnormalities, heart defects, hypotonia, talipes equinovarus, respiratory difficulties, developmental delay, and mental retardation [1–3]. Trisomy 5p is a chromosome 5p13 duplication syndrome (OMIM #613174).

Congenital diaphragmatic hernia (CDH) is a life-threatening disease with congenital defects of the diaphragm, and an incidence of 2–3 per 10,000 births [4]. Herniation of the intra-abdominal organs into the thorax leads to hypoplasia of the lungs and pulmonary hypertension. Most cases of CDH occur on the left side; bilateral CDH accounts for only 1% of all CDH cases [5]. The pathological causes of CDH remain unknown; however, increasing evidence shows its relationship to genetic or chromosomal abnormalities [6]. However, there are few studies on the association between trisomy 5p and CDH.

Here, we present a case of trisomy 5p diagnosed prenatally using ultrasound findings and amniocentesis, and bilateral CDH diagnosed postnatally.

## Case presentation

A 23-year-old primigravida Asian woman was referred at 33 weeks of gestation for several abnormal ultrasound findings, including small gastric bubble, talipes equinovarus, and polyhydramnios. On the first visit, the estimated fetal body weight was 1627 g (−1.5 SD), and the amniotic fluid index was 30.7 cm. The fetus had macroglossia, talipes equinovarus, and levocardia without cardiac structural abnormalities. The parents had no physical features and reported no past or family history. The mothers of the parents also had no history of miscarriages.

**Fig. 1:**
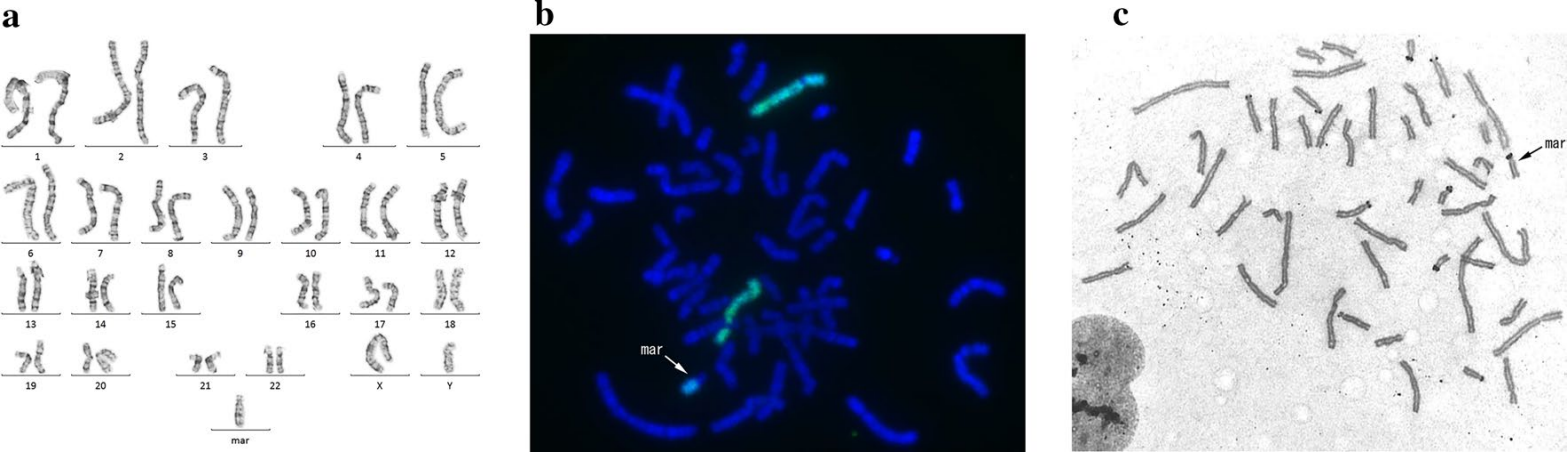
**a** The karyotype of amniocentesis as 47, XY, +mar. **b** Whole chromosome paint 5 revealed chromosome 5 and a positive marker chromosome (arrow). **c** Nucleolus organizer region showing a positive marker chromosome (arrow). Therefore, the marker chromosome was derived from chromosome 5 and the acrocentric chromosome, respectively.

The result of G-banding by amniocentesis performed at 32 weeks revealed a marker chromosome (Fig. 1a). Fluorescence *in situ* hybridization (FISH) was performed to examine the origin of the chromosome using whole chromosome painting (WCP) and nucleolus organizer region (NOR) probes. Both WCP5 (Fig. 1b) and NOR (Fig. 1c) were positive; therefore, the marker chromosome was determined to be derived from chromosome 5 and an acrocentric chromosome. From the G-banding findings, the derived region of chromosome 5 was pter→p13, and the karyotype was diagnosed as 47, XY, +mar. ish +mar(WCP5+), which included the critical region of 5p13 duplication syndrome. Array comparative genomic hybridization should be done to further confirm the origin of the marker chromosome, but consent could not be obtained.

**Fig. 2:**
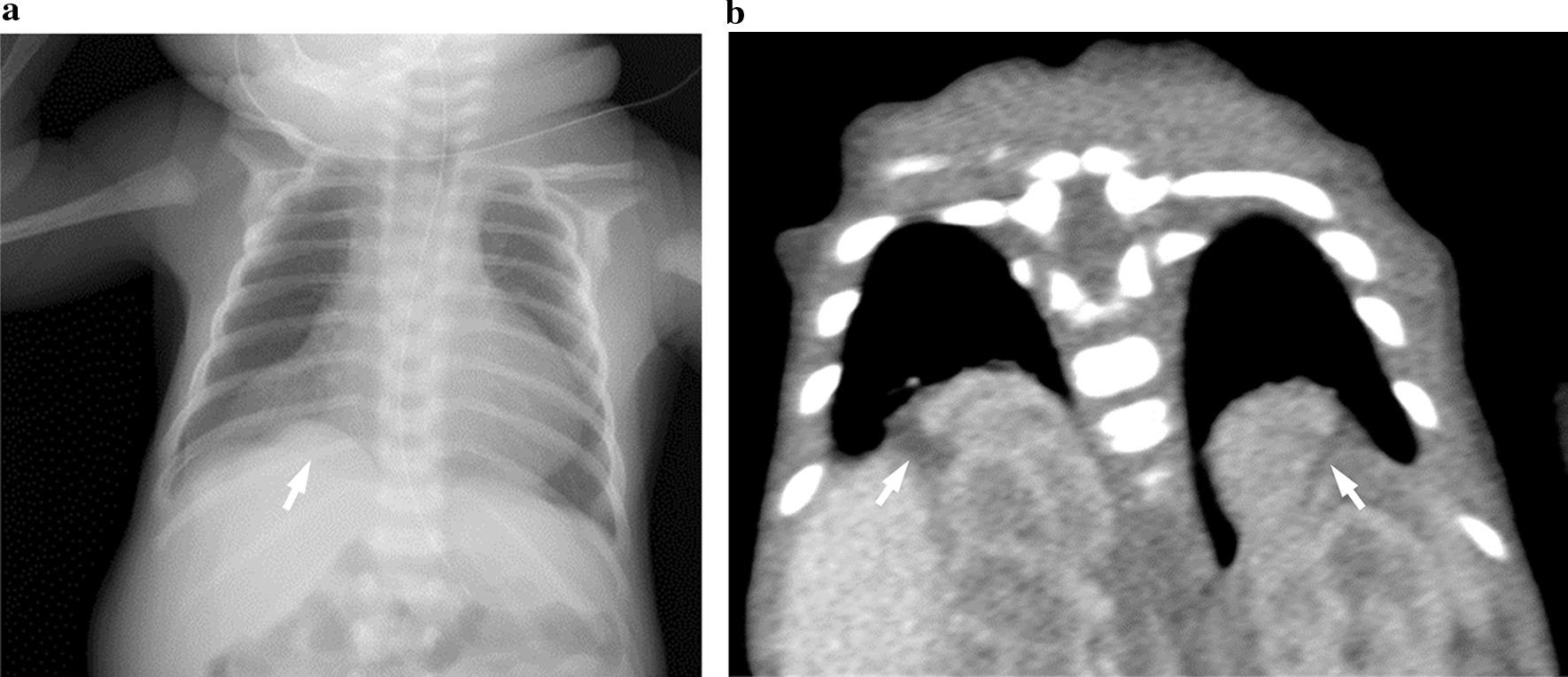
**a** Chest radiograph revealing a mass shadow (arrow) in the right lower lung lesion. **b** Computed tomography of coronal view. Arrows show bilateral herniation.

Amnioreduction was performed three times during the pregnancy, at 35, 38, and 39 weeks of gestation, for symptomatic polyhydramnios. At 39 weeks and 5 days, a 2462 g male infant was delivered after induction of labor. Immediately after birth, remarkable hypotonia was seen, and respiratory care was needed. APGAR scores at 1 and 5 minutes after birth were one and seven points, respectively. The infant’s physical features included enlarged head circumference, saddle nose, posterior neck thickening, low-set ears, macroglossia, cleft of the soft palate, micrognathia, and talipes equinovarus of both feet. After an examination of the infant, hypoplasia of the corpus callosum, atrial septal defect, and hypothyroidism were detected. On postnatal day 1, a mass shadow of the right lower lung field was detected through a chest X-ray (Fig 2a). Computed tomography was performed to confirm the mass and revealed a bilateral CDH (Fig. 2b). At 5 months after birth, deterioration of the respiratory condition due to laryngomalacia was managed by tracheotomy. At 8 months, cardioplasty and gastrostomy were performed for impaired swallowing function. During the operation, a defect was identified in the muscle of the medial-ventral diaphragm, and a membranous sac was formed in the right thorax. The liver and right adrenal gland were herniated into the right thorax, which may have caused the levocardia. However, only a medial muscle defect without herniation of organs was detected in the left side diaphragm. The bilateral pulmonary hypoplasia was mild, and the hernia sac simultaneously closed. At 10 months, the patient was discharged and transferred to a care hospital. The patient died of aspiration pneumonia and paralytic ileus at 17 months of age.

The postnatal karyotyping of peripheral blood was the same as that of prenatal karyotyping. Examination of their karyotype, especially regarding the presence of balanced reciprocal translocations, was offered to the parents. However, they declined the karyotyping examination.

## Discussion

The pre-and postnatal findings in the present case were consistent with previous reports excluding bilateral CDH [1–3]. A few studies have reported the pathogenic role of 5p in CDH [6]. Cornelia de Lange syndrome, which is caused by loss-of-function mutations of the *NIPBL* gene on 5p13, is also known to include CDH. A duplication on chromosome 5, including the *NIPBL* gene region, has been reported to occur in Cornelia de Lange syndrome [7]. Moreover, one case report presented a 5p deletion mosaic case with CDH [8]. These findings suggest that *NIPBL* might be one of the genes related to diaphragmatic genesis and development, although the pathological mechanism of CDH by *NIPBL* dysfunction remains unknown.

## Conclusion

In summary, we present a case of trisomy 5p diagnosed prenatally, and bilateral CDH detected after birth. Trisomy 5p and bilateral CDH are rare. Chromosome 5p might provide important clues clarifying the genetic causes of CDH; further research is required.

## Data Availability

Not applicable.
